# The Hahnemann Homœopathic Hospital, of Rochester, N. Y.

**Published:** 1889-12

**Authors:** 


### THE HAHNEMANN HOMŒOPATHIC HOSPITAL AT ROCHESTER, N. Y.

The building occupied as a hospital by the Hahnemann Homœopathists of Rochester was formerly a private residence. Built some years ago, it had been occupied by Judge Henry R. Selden and his family. Its location for an institution of this character is in many respects unsurpassed.

The grounds, some three acres in extent, are in the high degree of cultivation common to places owned by gentlemen of wealth and leisure.

But the point that especially commends the spot to invalids is the healthfulness of its situation.

Placed upon high land, overlooking the city, where are few residences to impair the purity of its surroundings, it impresses the most casual visitor with the delightfully invigorating quality of its atmosphere. This seems charged with health-giving properties, while the soot, dust, and sewer gas of the city seem never to approach.

During the winter and early spring the workmen employed in renovating the premises frequently commented upon the good effects they themselves received from this source.

Another excellence to be mentioned is the restfulness of the place. The thousand noises that banish sleep and make life a torture to sensitive nerves are lost before they reach this spot, and the quiet peacefulness of a Sabbath in the country reigns continually.

The house, being "large and roomy," has been changed into a hospital at small expense. Upon the first floor are the reception-room, doctor's office, a room for surgical operations, one male ward, a dining-room, bath-room, and toilet-room, with a wide hall running through the house.

On the second floor there are two wards for women, four rooms furnished for private patients, two other rooms that may eventually be furnished for patients, and a bath-room. The third floor is now being fitted up for use of the various assistants necessary to such an institution. In the basement are the kitchen, laundry, and storerooms.

The building was formally opened for occupancy, April 10th of the present year, by the Board of Managers, who invited such of the public as were interested in the enterprise to listen to some addresses, partake of refreshments, and wish them "Godspeed" in their enterprise. Dr. J. A. Biegler made a short speech, and Dr. W. T. Brownell introduced the speaker of the evening, Dr. Clarence Willard Butler, of Montclair, N. J. This gentleman made an appeal for Hahnemann's Homœopathy. His words were fitly spoken, and gave great pleasure to all who were so fortunate as to hear them. More than two hundred guests were present, and the Hahnemann Homœopathic Hospital began its career of a usefulness which, it is to be hoped, will increase more and more until all shall acknowledge the truthfulness of its principles and the charm of its practice.

The ladies, always forward in every good work, are laboring energetically to build up and to sustain this hospital. They have begun the publication of a paper to be known as the *Hahnemann Advocate*, to aid the good work. The price of the *Advocate* is only fifty cents a year; address is 19 Grove Place, Rochester, N. Y.

The Hahnemann Homœopathic Hospital is now established on such a firm and permanent footing that Dr. J. A. Biegler, who has from its commencement served as chief of staff, feels that he can with propriety carry out his expressed declaration when accepting the position and file his resignation. He is succeeded by Dr. Allen B. Carr, whose acceptance is assurance that nothing will be wanting in the future, as nothing has been wanting in the past, for the success of the institution. In resigning Dr. Biegler does not relinquish one iota of his interest in the Hospital. The step has been rendered necessary by his pressing professional engagements and duties in private practice, and other circumstances of a nature personal to himself. He will remain as consulting physician, and will in every way aid his successor and associates. Before resigning he made sure of the permanent occupancy and ultimate ownership of the property, free from all obligations, by the Society.

